# Characterization of Arils Juice and Peel Decoction of Fifteen Varieties of *Punica granatum* L.: A Focus on Anthocyanins, Ellagitannins and Polysaccharides

**DOI:** 10.3390/antiox9030238

**Published:** 2020-03-13

**Authors:** Diletta Balli, Lorenzo Cecchi, Mohamad Khatib, Maria Bellumori, Francesco Cairone, Simone Carradori, Gokhan Zengin, Stefania Cesa, Marzia Innocenti, Nadia Mulinacci

**Affiliations:** 1 Department of Neuroscience, Psychology, Drug and Child Health, Pharmaceutical and Nutraceutical Section, University of Florence, 50019 Florence, Italy; diletta.balli@unifi.it (D.B.); lo.cecchi@unifi.it (L.C.); mohammadtttt@yahoo.com (M.K.); maria.bellumori@unifi.it (M.B.); marzia.innocenti@unifi.it (M.I.); nadia.mulinacci@unifi.it (N.M.); 2 Department of Drug Chemistry and Technology, University “La Sapienza” of Rome, 00185 Rome, Italy; francesco.cairone@uniroma1.it (F.C.); stefania.cesa@uniroma1.it (S.C.); 3 Department of Pharmacy, University “G. d’Annunzio” of Chieti-Pescara, 66100 Pescara, Italy; 4 Department of Biology, Selcuk University, 42130 Konya, Turkey; gokhanzengin@selcuk.edu.tr

**Keywords:** pomegranate, HPLC–DAD–MS, phenolic compounds, size exclusion chromatography, CIEL*a*b*, enzyme inhibitory activity

## Abstract

Pomegranate is receiving renewed commercial and scientific interest, therefore a deeper knowledge of the chemical composition of the fruits of less studied varieties is required. In this work, juices from arils and decoctions from mesocarp plus exocarp were prepared from fifteen varieties. Samples were submitted to High Performance Liquid Chromatography—Diode Array Detector–Mass Spectrometry, spectrophotometric and colorimetric CIEL*a*b* analyses. Antioxidant, antiradical and metal chelating properties, inhibitory activity against tyrosinase and α-amylase enzymes were also evaluated. All varieties presented the same main phenols; anthocyanins and ellagitannins were widely variable among varieties, with the richest anthocyanin content in the juices from the Wonderful and Soft Seed Maule varieties (approx. 660 mg/L) and the highest ellagitannin content in the peel of the Black variety (approx. 133 mg/g dry matter). A good correlation was shown between the colour hue and the delphinidin/cyanidin ratio in juices (*R*^2^ = 0.885). Total polysaccharide yield ranged from 3% to 12% of the peels’ dry weight, with the highest content in the Black variety. Decoctions (24.44–118.50 mg KAE/g) showed better in vitro antioxidant properties and higher inhibitory capacity against tyrosinase than juices (not active-16.56 mg KAE/g); the inhibitory capacity against α-amylase was similar and quite potent for juices and decoctions. Knowledge about the chemical composition of different pomegranate varieties will allow for a more aware use of the different parts of the fruit.

## 1. Introduction

Pomegranate (*Punica granatum* L.) belongs to the Punicaceae family and is classified among the top seven fruits with the highest beneficial properties for human health [1,2]. Some of these beneficial properties concern the treatment or the prevention of thyroid disorders, bronchial asthma, diarrhea, arterial pressure disorders and cardiovascular diseases [3,4,5]. Its scientific name derives from the Latin *pomum* (apple) and *granatus* (grainy), meaning seeded apple. Pomegranate is native to central Asia, notably Iran. Nowadays, it is a very widespread plant that grows in many different geographical regions including the Mediterranean, Asia and California [6]; indeed, the tree is highly adaptive to a wide range of climates and soil conditions including arid regions. Iran and India, but also North and South America and Europe, particularly the Mediterranean regions, are the leading producers [6,7].

Pomegranate is a fleshy berry (approx. 6.2–12.5 cm wide, and 200–650 g in weight) with varying colour from reddish-yellow to green with reddish zones. Differences are established according to potential market and consumer preferences, taking into account parameters such as fruit size, husk and aril colors, seed hardness, ripening level, juice content, acidity, sweetness and astringency. Nowadays, scientific interest in the chemical characterization of pomegranate is focused not only on the edible parts (arils), but also on the inedible ones, such as the peel, which accounts for 40–50% of the whole fresh fruit by weight. This part of the fruit is generally discarded, and thus constitutes the main by-product obtained after juice extraction [7].

Pomegranate fruit is known as a good source of phenolic compounds such as ellagic acids, and several hydrolysable tannins (mainly ellagitannins, such as punicalagins and punicalins, and numerous gallagyl esters). Anthocyanins, consisting mainly of cyanidin and delphinidin glucosides, are characteristic of the juice, although these pigments are present not only in the arils but also in the peel at different percentages [8,9]. Fresh peel contains high percentages of water (around 70–75%), while the dry material is composed of simple sugars (30–35%), phenolic compounds (10–20%) and polysaccharides (10–15%) [8,9,10,11].

The polysaccharides of *Punica granatum* L. can be included among the bioactive components of the fruit along with ellagitannins. Recently, beneficial effects related to pomegranate polysaccharides, such as antioxidant and immunomodulatory properties [3,12,13] and in vitro prebiotic properties [10], have been pointed out. Furthermore, the combination of polysaccharides and ellagitannins has shown the ability to counteract the initial, intermediate and late phases of colon carcinogenesis in rats [14]. So far, the total content and the chemical features of the polysaccharides from pomegranate have not been fully explored, despite the increased relevance and economic importance of this fruit and of its by-products. According to the literature, [2,10,15,16,17,18] pectins are reported as the main polysaccharide constituents, however, the term pectin refers to a complex mixture of polysaccharides with different structures and chemical-physical properties, as summarized in a recent review [19]. Hot water was recently proposed to extract pectin from pomegranate peel [16], and it was demonstrated that a boiling treatment did not affect the molecular weight as determined by size exclusion chromatography [10]. More generally, knowledge about pomegranate polysaccharides is, so far, limited, particularly in relation to a specific varieties.

European regulations do not allow much waste to be disposed of directly in landfills because this can pose a risk to the environment [20]. On the other hand, a suitable use of such residue can help to promote innovation in the agro-processing sector [18,19].

Given the increased interest in pomegranate fruit, knowledge of the chemical constituents extractable from both arils and peel is desirable to valorize specific cultivars, among the approximately 500 present worldwide [6]. An increased amount of bioactive compounds in a specific variety, typically anthocyanins in arils and ellagitannins and polysaccharides in peel, can be a useful tool to favor the cultivation of new varieties and the reuse of the juices by-products.

The aim of this work was the characterization of fifteen pomegranate varieties grown in the same hatchery, some of them never studied so far, to shed light on the composition of the fruits. To avoid environmental interference, all pomegranate samples came from the same harvesting year (2017) and the fruits were collected at complete maturation. Anthocyanins and ellagitannins from arils and polysaccharides and ellagitannins from peel were characterized. The obtained polysaccharides from some selected varieties were characterized by Size Exclusion Chromatography (SEC); some fractions were isolated, and some of these were selected and submitted to sugar analysis. CIEL*a*b* analysis was applied to reveal, by a simple colorimetric assay, any quali-quantitative difference among samples. Color measurement plays a crucial role in those functional foods, such as pomegranate, in which bioactive molecules are intensely colored. Moreover, the color expressed by a foodstuff is undoubtedly associated with its genuineness, and additionally with its specific chemical profile, which, in turn, depends on ripening stage and cultivar. The possibility of evaluating this organoleptic character without any treatment and with a very simple, fast and cheap method can provide a lot of useful information to complement that deriving from the quali-quantitative analysis carried out by chromatographic methods. The antioxidant properties were also evaluated for aril juices and peel decoctions by a pool of in vitro tests. Finally, juices and decoctions were used to study their biological properties by measuring their inhibition against two enzymes, α-amylase and tyrosinase.

## 2. Materials and Methods

### 2.1. Samples

The fifteen pomegranate fruits were collected from the same plant nursery (Latina, Italy) and selected by an expert plant breeder from a collection of over 200 different pomegranate varieties. The whole fruits were divided into arils and peel (mesocarp + exocarp) and the fresh tissues were stored at –22 °C until the extractive procedure and juice preparation.

### 2.2. Reagents

Ultrapure water was obtained from a Milli-Q-system (Millipore SA, Molsheim, France). All solvents of analytical HPLC grade were obtained from Sigma-Aldrich (St. Louis, MO, USA). Ellagic acid (purity ≥ 95%) and punicalagin (purity ≥ 98%) were purchased from Sigma Chemical Co. (St. Louis, MO, USA), and oenin chloride (purity ≥ 95%) was purchased from Extrasynthese (Genay, France). Dextrans of different molecular weights (MWs: 2000, 1100, 410, 150, 50 kDa) and the sucrose (360 Da) used for SEC were obtained from Sigma-Aldrich (St. Louis, MO, USA).

### 2.3. Juice Production

The arils of each sample were divided into three portions, each treated separately by the Hurom extractor (HU-700) using a rapid process at low temperatures to obtain three replicates of juices. Each sample was diluted (1:1 *v*/*v*) with ethanol (2% HCOOH), centrifuged at 14,000 rpm for 5 min, and the recovered supernatant was analyzed by HPLC–DAD–MS.

### 2.4. Decoction from Peel (Mesocarp + Exocarp)

The fresh mesocarp and exocarp were used to prepare a decoction according to Khatib et al. [10]: briefly, 5 g of pomegranate peel was boiled for 1 h in 200 mL of water. The sample was cooled at room temperature and centrifuged (4500 rpm, 8 min, 4 °C) and the supernatant was brought to a volume of 200 mL (exactly measured) with distilled water. An aliquot of 10 mL was withdrawn and used for HPLC–DAD analysis of ellagitannins, colorimetric measurements, spectrophotometric determinations and enzymatic tests.

Polysaccharide precipitation was performed as suggested by Khatib et al. [10], by consecutive additions of several aliquots of ethanol (100 mL each). After each ethanol addition and the consequent precipitation of polysaccharides, the obtained mixture was centrifuged (4500 rpm, 8 min, 4 °C), and the solid phase containing polysaccharides was collected, freeze-dried and weighed for calculation of yields. The precipitation of polysaccharides was completed after addition of three aliquots of alcohol (i.e., the 4th addition of ethanol did not allow any further precipitation of polysaccharides); thus, we collected three polysaccharide fractions for each sample (DP-1, DP-2 and DP-3).

### 2.5. HPLC–DAD Analyses of Juices and Decoctions

Ellagitannins and anthocyanins were analyzed using a HP 1200L liquid chromatographic system equipped with a degasser, a column oven, a binary pump and a diode array detector (all from Agilent Technologies, Palo Alto, CA, USA). For the analysis of both types of phenols, the Kinetex, 100, EC-C18 (30 × 3 mm, 2.6 µm, Agilent, Palo Alto, CA, USA) column was employed using CH_3_CN (solvent A) and H_2_O acidified by 3% *v/v* HCOOH (solvent B) with the following linear gradient as the elution method: solvent A varied from 5% to 25% over 8 min, then remained at 25% for 10 min; then, in 2 min, solvent A reached 95% and was kept in this condition for 6 min, for a total analysis time of 28 min; oven temperature 25 °C, equilibration time 10 min, flow rate 0.4 mL/min. The injection volume was 2 μL for ellagitannins extracts (decoctions) and 10 μL for anthocyanins extracts (centrifuged juices). The chromatograms were recorded at 330, 370, 380, 520 nm.

Two five-point linear calibration curves were built and used for quantitation of ellagitannins: one at 380 nm using the racemic mixture of α- and β-punicalagins (purity ≥ 99%) as a standard, linearity range 0–5 µg (*R*^2^ = 0.998); the other one at 370 nm using ellagic acid (purity 95%) as a standard, linearity range 0–1.7 µg, (*R*^2^ = 0.999). Each ellagitannin was thus quantified using either one or the other calibration curve according to its λ_MAX._ The anthocyanins were quantified at 520 nm using a calibration curve built using oenin chloride (purity ≥ 95%) as a standard, linearity range 0–2.6 µg (*R*^2^ = 0.999).

### 2.6. HPLC-MS Analysis

Decoctions and juices were analyzed on a quadrupole ionic trap LTQ (Thermo Finnigan, San Jose, CA, USA) coupled to an HPLC (Thermo Finnigan Surveyor, San Jose, CA, USA); the HPLC conditions were the same used for the HPLC–DAD analysis. The analyses were conducted with the following ESI parameters (electrospray ionization): Sheath Gas Flow Rate: 35; Aux Gas Flow Rate: 10; Sweep Gas Flow Rate: 7 (the software reports all the gas flows in arbitrary units). Spray Voltage was 4.20 V; Capillary Temperature was 280 °C; Capillary Voltage was –23 V and Tube Lens was –53 V. Acquisition for mass analysis was performed in negative and positive ion mode, in a full-spectrum scan in the range of *m*/*z* from 100 to 1800.

### 2.7. Analysis of Polysaccharides by Size Exclusion Chromatography (SEC)

Polysaccharides of four selected samples, namely Acco, Black, Wonderful and Mollar de Elche, were analyzed by SEC to determine the apparent molecular weight (hydrodynamic volume) of their main constituents. Briefly, the freeze-dried samples were dissolved in distilled water at a final concentration close to 0.5 mg/mL. The samples were analyzed according to a procedure described by Chamizo et al. [21]. Briefly, two columns (Phenomenex, Torrance, CA, USA), both of 700 mm length and 7.8 mm internal diameter, were used in series: PolySep-GFC-P 6000 (separation range from 100 kDa to 15 MDa) and PolySep-GFC-P 4000 (separation range from 0.3 to 400 kDa); the instrument was a ProStar HPLC Chromatograph (Varian, Palo Alto, CA, USA) coupled with a refractive index detector (mod 355). HPLC-grade water was used as the eluent with a flow of 0.6 mL/min and a total analysis time of 70 min. Blue dextrans of various molecular weights (50–2000 kDa) were used as internal standards to determine the hydrodynamic volume.

### 2.8. Sugars Analysis

The second fractions (D-P2), obtained after the second addition of 100 mL of ethanol to the decoctions (paragraph 2.4) of the Black and Wonderful varieties, were dialyzed (cut-off 12–14 kDa), freeze-dried and treated according to Nunes & Coimbra [22] for the determination of neutral sugars after acid hydrolysis and conversion to the corresponding alditol acetates. Gas chromatography was performed with a 5890 instrument using a FID detector (Hewlett-Packard, Palo Alto, CA, USA), with a split ratio of 1:60. A CP-Sil-43 CB column (Chrompack, Middelburg, Holland), 25 m × 0.15 mm i.d., 0.20 μm film thickness, was used. A temperature of 220 °C was applied to the injector and detector; the following gradient was used with a rate of 0.5 °C/min: 180 °C for 5 min, then 200 °C for 20 min; linear velocity of the carrier gas (H_2_) was set at 50 cm/s at 200 °C. Furthermore, uronic acids were colorimetrically determined using *m*-phenylphenol as previously reported [22].

### 2.9. Colorimetric CIEL*a*b* Analysis of Juices and Decoctions

The color expressed by the samples was measured by a colorimeter X-Rite SP-62 (X-Rite Europe GmbH, Regensdorf, Switzerland). Six different measurements were randomly performed by the instrument on the cell surface; the reflectance curves were registered and the color was expressed using the CIEL*a*b* coordinate system in which L*, a* and b* resemble respectively the lightness, the red or green, the yellow or blue color, as defined by the “Commission Internationale del Eclairage” (CIE): the two derived parameters C*_ab_ and h_ab_ were calculated as previously reported [23]. Samples of pomegranate juices and decoctions, obtained as previously described, were used as such to re-fill the quartz cell for fluid measurements. Each experiment was performed four times and the results are expressed as the mean value ± standard deviation (SD).

### 2.10. Total Phenolic Content (TPC) Evaluation

The TPC was determined using the Folin–Ciocâlteu method [24,25]. A mixture containing 20 µL of each extract (1 mg/mL), 100 µL of Folin-Ciocâlteu reagent (diluted 1:9 in distilled water) and 80 µL of sodium carbonate solution (2%) was incubated in the dark for 30 min at room temperature. Successively, the absorbance of each sample was recorded at 760 nm, using gallic acid as a reference standard (calibration curve from different concentrations: 0–5 µg; Absorbance: 0.2163x (µg gallic acid), *R*^2^ = 0.9962). The TPC was determined as gallic acid equivalent (GAE) in mg/g dry weight (dw) of extract.

### 2.11. Total Flavonoid Content (TFC) Evaluation

The TFC was determined using the procedure described by Mocan et al. [26]. An amount of 100 µL of 2% AlCl_3_ aqueous solution was added to 100 µL of juice or decoction samples (1 mg/mL). After 15 min of incubation, the absorbance of each sample was recorded at 420 nm. Rutin was used as a reference standard (calibration curve from different concentrations: 0–20 µg; Absorbance: 0.1301x (µg rutin) + 0.0506, *R*^2^ = 0.9942) in order to express the TFC as rutin equivalent (RE) in mg/g dry weight of extract.

### 2.12. 2,2-Diphenyl-1-Picrylhydrazyl Radical Scavenging Assay

The scavenge ability of the free radical 2,2-diphenyl-1-picrylhydrazyl (DPPH) was described by Martins et al. [27]. The mixture included 30 µL of sample solution (1 mg/mL) and the solution of DPPH in methanol. After 30 min of incubation in the dark, DPPH radical reduction was recorded at 515 nm. Trolox was chosen as a reference (calibration curve from different concentrations: 0–5 µg), Absorbance: 14.058x (µg trolox) + 1.932, *R*^2^ = 0.9948) and the results were reported as Trolox equivalents (TE) per g of dry weight of extract.

### 2.13. Trolox Equivalent Antioxidant Capacity (TEAC) Assay

The radical scavenging activity of juices and decoctions toward the 2,2′-Azino-bis(3-ethylbenzothiazoline-6-sulfonic acid (ABTS) radical cation was evaluated according to Mocan et al. [24]. The final results were expressed by means of plotting a Trolox calibration curve (calibration curve from different concentrations: 0–2.5 µg), Absorbance: 12.054x (µg trolox) – 2.7398, *R*^2^ = 0.9674), as millimoles of Trolox equivalents (TE) per gram of dry extract (mmol TE/g dw extract).

### 2.14. Ferric Reducing Antioxidant Power Assay

In this assay, the reduction of Fe^3+^-TPTZ (2,4,6-tris(2-pyridyl)-*s*-triazine) to Fe^2+^-TPTZ was recorded following Damiano et al. [28]. The reaction mixture (25 µL of each sample (1 mg/mL) and 175 µL of the Ferric Reducing Antioxidant Power (FRAP) reagent) was incubated at room temperature in the dark for 30 min and the absorbance of each solution was read at 593 nm. The results were reported as millimoles of Trolox equivalents (TE) per gram of extract (mmol TE/g extract) (calibration curve from different concentrations: 0–2.5 µg; Absorbance: 0.3178x (µg trolox) + 0.0053, *R*^2^ = 0.9994).

### 2.15. CUPRAC Assay

The cupric reducing antioxidant capacity (CUPRAC) method was carried out by adding 0.5 mL of each sample (1 mg/mL) to a reaction mixture containing 1 mL each of neocuproine (7.5 mM), CuCl_2_ (10 mM), and NH_4_Ac buffer (1 M, pH 7.0). After 30 min of incubation at room temperature, the absorbance of each solution was read at 450 nm. The results were reported as millimoles of Trolox equivalents (TE) per gram of extract (mmol TE/g extract) (calibration curve from different concentrations: 0–2.5 µg; Absorbance: 0.1317x (µg trolox) + 0.0068, *R*^2^ = 0.9968) [29].

### 2.16. Phosphomolybdenum Assay

The total antioxidant capacity of the samples was measured using a phosphomolybdenum assay. Briefly, 0.3 mL sample solution (1 mg/mL) was added to 3 mL of reagent solution containing 4 mM ammonium molybdate, 28 mM sodium phosphate, and 0.6 M sulfuric acid. The absorbance was then read at 695 nm after 90 min of incubation at 95 °C against a blank. The results were reported as millimoles of Trolox equivalents (TE) per gram of extract (mmol TE/g extract) (calibration curve from different concentrations: 0–100 µg; Absorbance: 0.061x (µg trolox) + 0.0056, *R*^2^ = 0.9983) [30].

### 2.17. α-Amylase Inhibitory Activity

α-Amylase inhibition was assayed using a reaction mixture of 25 µL of sample solution (1 mg/mL) and 50 µL of α-amylase solution (from porcine pancreas, 10 U/mL) dissolved in phosphate buffer (pH 6.9 containing 6 mM NaCl). After 10 min of incubation at 37 °C, 50 µL of starch solution (0.05%) was added. Hydrochloric acid (1 M, 25 µL) was then added to stop the reaction, followed by the addition of 100 µL of iodine-potassium iodide solution. All the absorbances were measured at 630 nm after further 10 min of incubation at 37 °C. The results were reported as millimoles of acarbose equivalents (ACAE) per g of extract (mmol ACAE/g extract) (calibration curve from different concentrations: 0–50 µg; Absorbance: 2.0654x (µg acarbose) + 4.0458, *R*^2^ = 0.9543) [30].

### 2.18. Tyrosinase Inhibitory Activity

Tyrosinase inhibitory activity of samples was determined by a method previously described by Masuda et al. [31]. For each sample (dissolved in water containing 5% DMSO, 1 mg /mL) four wells of 200 µL, designated as A, B, C, D, contained: (A) 140 µL of 66 mM phosphate buffer solution (pH = 6.6) (PBS), 40 µL of mushroom tyrosinase in PBS (23 U/mL) (Tyr), (B) 160 µL PBS, (C) 80 µL PBS, 40 µL Tyr, 40 µL juice or decotion sample and 80 µL PBS and (D) 120 µL PBS and 40 µL sample. The plate was then incubated for 10 min at room temperature, and then 40 µL of 2.5 mM L-DOPA in PBS solution was added in each well and the mixtures were further incubated for 20 min at room temperature. The results, deriving from the absorbance of each well measured at 475 nm, were reported as mg kojic acid equivalents per gram of dry weight of extract (mg KAE/g extract) using kojic acid as a reference compound (calibration curve from different concentrations: 0–0.3 mg; Absorbance: 1.5235x (mg kojic acid) + 0.0055, R^2^ = 0.9981).

### 2.19. Statistical Analysis

The results from triplicate analyses were expressed as mean ± SD using EXCEL software (version 2013) (Redmond, Washington, USA) in-house routines. One-way ANOVA followed by Tukey’s multiple range was done to investigate significant differences (*p* < 0.05) between the tested samples, and the statistical process was performed using GraphPad Prism 8.0 program (GraphPad Software, San Diego, CA, USA).

## 3. Results and Discussion

The fifteen varieties listed in Table 1 were selected by a plant breeder from over 200 different genotypes collected in the same plant nursery. These samples include varieties widely used to produce pomegranate juices, such as Wonderful throughout Europe, Mollar de Elche in Spain, and the early variety Acco from Israel [7]. Other samples are characterized by the softer consistency of the seeds (Soft Seed Maule^®^ 116/17), the large size of the fruit (Grossa di Faenza), or the non-pigmented arils and the intense red-blue color of the skin (Black). Concerning the percent of the peel in the fresh fruit weight, the range was from 41.6% for the Austin variety up to 68% for the Shirin Zigar variety, which shows the maximum thickness of peel (0.7 cm). As shown in Table 1, most of the samples have a peel weight close to the 50% of the fruit. As for the use of pomegranate peel, the major by-product of juice production, the literature so far does not mention the varieties used for the technological applications, and the only available data are usually for the Wonderful variety because it is the most cultivated in the world [11,18]. To the best of our knowledge, most of the other varieties listed in Table 1 have never been previously studied.

### 3.1. Juices and Decoctions

In this work, we propose the use of the same chromatographic method for the analysis of both the main ellagitannins and anthocyanins in juices and decoctions, in order to provide a simple and easily applicable elution method for quality control of the samples. Both the decoctions and the juices of all the samples present almost superimposable HPLC–DAD profiles, with only negligible differences, mainly regarding some minor compounds. As an example, we reported in Figure S1 the profiles obtained at 370 nm, 380 nm and 520 nm for decoction of the Black variety and for the juice of Wonderful variety.

We identified nine main molecules, including ellagitannins, such as ellagic acid and α- and β-punicalagins; five anthocyanins were found, particularly delphinidin-3,5-*O*-diglucoside, cyanidin-3,5-*O*-diglucoside, delphinidin-3-*O*-glucoside, cyanidin-3-*O*-glucoside and pelargonidin-3-*O*-glucoside, in agreement with the previous literature on pomegranate [8]. The identification was performed according to the MS analysis, UV-Vis spectra, retention time and comparison with available commercial standards (Table S1).

**Figure 1:**
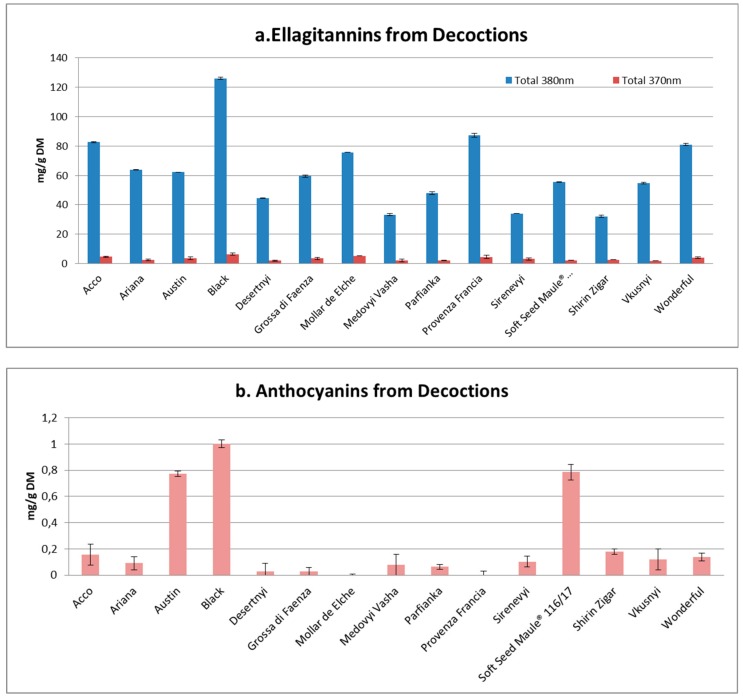
Ellagitannins (**a**) and anthocyanins (**b**) from decoctions. Ellagitannin content is expressed as mg of punicalagin on g of dried matter when the λ_MAX_ is close to 380 nm and as mg of ellagic acid on g of dried matter when the λ_MAX_ is close to 370 nm. The content of anthocyanins is expressed as mg of oenin on g of dried matter.

On the contrary, significant and, in some cases, large differences were observed between the phenolic contents of the different decoctions and juices of the fifteen varieties. The decoctions showed greater amount of total ellagitannins and a lower concentration of anthocyanins, with only a few exceptions represented by those varieties characterized by a darker skin color as Black, Austin and Soft Seed Maule^®^ 116/17 (Figure 1).

**Figure 2:**
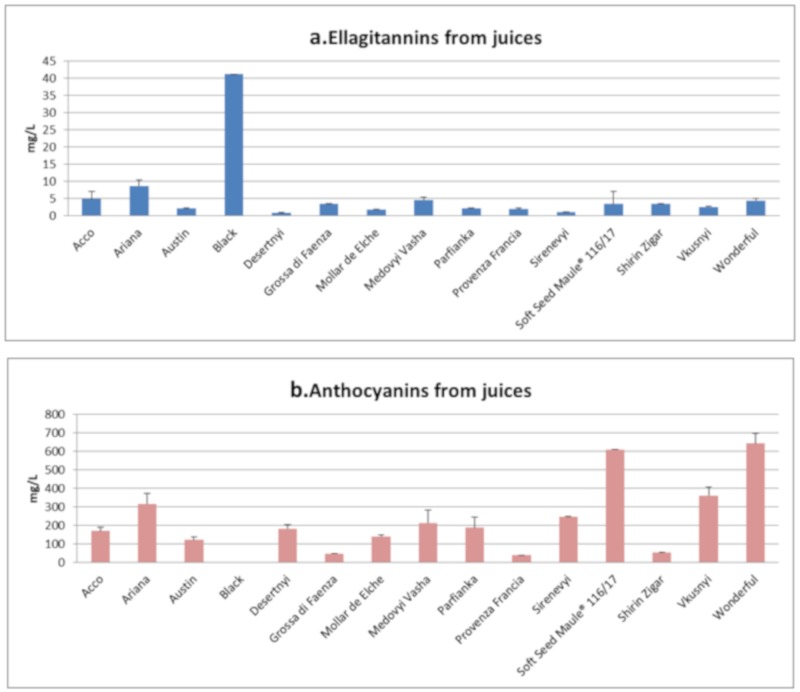
Ellagitannins (**a**) and anthocyanins (**b**) from juices. Ellagitannins content is expressed as mg of ellagic acid on g of dried matter (all the detected ellagitannins showed λ_MAX_ close to 370 nm). The content of anthocyanins is expressed as mg of oenin on g of dried matter.

Juices were poor in ellagitannins and rich in anthocyanins, with the highest content of this latter class in the Soft Seed Maule^®^116/17 and Wonderful samples, both with values close to 600 mg/L (Figure 2). This result is noteworthy, since it is well-known that when the concentration is too high, ellagitannins can determine an unpleasant taste in juices, due to their interactions with the salivary proteins, also typical of other tannins. The result was not unexpected because our juices were obtained only using the arils, recognized as a poor source of ellagitannins [8]. Finally, Black was the unique variety with white arils, and was therefore characterized by the absence of anthocyanins (Figure 2b); the juice of this variety showed an appreciable quantity of ellagitannins, about ten times higher than the average value measured for the other varieties (Figure 2a).

Phenolic composition usually varies among pomegranate cultivars depending on environmental conditions, such as geographic location, fruit maturity stage, peel and aril color. For this reason, there is a wide range in phenolic content among different pomegranate fruits, as reported by the review of Singh et al. [9], in which several samples collected in different countries were compared, but information on specific varieties is lacking.

Concerning our samples, a detailed distribution of all the detected ellagitannins and anthocyanins in the fifteen varieties is shown in Table 2 and Table 3, for decoction and juices respectively. For the decoctions, the amount of ellagic acid in all the samples was low, with the highest concentrations at 0.4 mg/g dried peel, in Mollar de Elche and Provenza Francia. The sum of α- and β-punicalagins, reported as the main compounds in pomegranate peel [8], ranged from 13 to 105 mg/g; in 13 of the 15 pomegranate samples, the sum of the two punicalagins comprised approx. 70–80% of the total ellagitannin content.

The juices were characterized by the presence of several anthocyanins as the main phenolic components, with strong differences concerning the relative amount of each single anthocyanin glycoside. Among these pigments, the delphinidin-3,5-*O*-diglucoside could be suggested as a marker to build a variety fingerprint because it is the only metabolite not detected in all the samples (Table 3). Unlike in decoctions, the α- and β-punicalagins were not found in the juices of all the varieties (the only exception was Black). Most of the commercial pomegranate juices are usually prepared by pressing half of the whole fruit, which leads to a co-extraction of some ellagitannins from peel [8]. In our work we used only arils for producing juices.

Concerning polysaccharides, Table 4 shows the yields (% of dry peel) for the three fractions obtained from each variety. Data show that the second fractions (D-P2) contained the greatest amount of polysaccharides in almost all varieties, while the sum of the amounts of polysaccharides in the three collected fractions ranged from 3% to 12% of the dry weight of the skin. A certain variability was observed among the samples, with the total amount in Wonderful, which was comparable to previous literature data [10] and in percentages similar to those of Sirenevyi, Medovyi Vahsha, Mollar de Elche, Soft Seed Maule^®^ 116/17 and Parfianka (Table 4).

### 3.2. Size Exclusion Chromatography and Sugar Analysis

Four samples (Acco, Black, Wonderful and Mollar de Elche) within the fifteen varieties were selected, to further investigate the apparent molecular size of polysaccharides and sugar composition. These samples were selected in light of the greatest morphological differences between their fruits, and because these varieties are the most represented on the market. Results from SEC (Figure S2) indicate an apparent molecular weight of approx. 2 million Dalton for the main polysaccharides in Acco, Black, Wonderful and Mollar de Elche, with low percentages of oligosaccharides (<3 K), which presumably remained trapped in the polymer structure during the polysaccharides’ precipitation.

**Figure 3:**
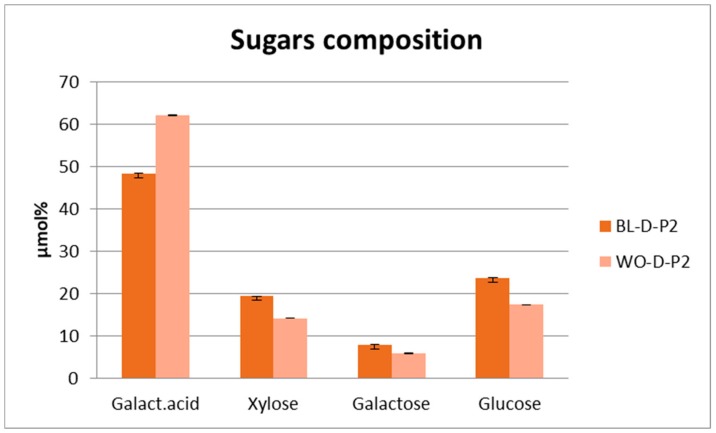
Sugar composition of the polysaccharide fraction (D-P2) of Black and Wonderful varieties. Data are expressed in µmol (%) as a mean of three independent measurements.

Sugar composition was preliminarily determined for the main fractions of the Wonderful and Black varieties. To this end, the second fraction (D-P2) of the two varieties was dialyzed and hydrolyzed to determine the content of neutral sugars and galacturonic acid. Despite the great morphological differences between the fruits of these two varieties, the results highlighted a similar sugar composition, with galacturonic acid and glucose as the main sugars. The high percentages of galacturonic acid in the total sugars are in agreement with the presence of pectin as a constituent of these fractions, and the absence of rhamnose implies that pectins present in the samples do not belong to the group of rhamnogalacturonan I and II (Figure 3).

This result, concerning the sugar composition of the polysaccharide in fraction 2 (D-P2), suggests that even varieties of the fruit with different shapes and morphological aspects are characterized by the presence of similar pectin structures. Further studies should be performed to better clarify the chemical structure of pectin in pomegranate fruits and evaluate their properties and suitability for specific technological purposes in formulation of foods or for cosmetic applications.

### 3.3. Colorimetric CIEL*a*b* Analysis

As is well known, different pigments deeply characterize pomegranate fruit components: anthocyanins, contained in arils, confer a red brilliant color, and yellow ellagitannins are represented in both the fruit pulp and peels, contributing to or determining their color. Historically, natural colorants have been extracted from agricultural residues and pomegranate husks; in particular, they represented a matrix largely used for natural dying for their good properties of fastness and color strength and depth [32]. More recently, increasing and renewed interest has addressed the pigment content of fruit and vegetables for its implication in health processes [33].

Therefore, in addition to being the first character that consumers evaluate when they choose any product, color represents a real fingerprint of a sum of chemical properties exerted by a foodstuff. Moreover, the adopted analytical technique allows performing color detection on samples as such, in the form of powder, homogenates or suspensions, not requiring any kind of pretreatment [4,34,35].

The pomegranate juices and decoctions, obtained and analyzed as described above, were submitted to colorimetric CIEL*a*b* analysis to evaluate how the different varieties could influence the final expressed color and if some conclusions could be drawn about the correlation among colorimetric and HPLC data. The CIEL*a*b* system is based on three parameters: L* (luminance), a* (greenness for negative values and redness for positive values) and b* (blueness for negative value and yellowness for positive values). The combination of a* and b* values will give the same color saturation (C*_ab_) and tonality (h_ab_), whereas the perceived color will depend also by the expressed luminance.

Results obtained for pomegranate decoctions (Table 5) showed L* values between 40.18 and 47.64 in a quite narrow range, whereas a* falls between 0.70 and 10.92, and b* between 2.54 and 23.48, showing significant differences among samples, with color ranging from yellow to brown. Actually, it is not possible to match the obtained colorimetric data with that deriving from the yellow ellagitannins quantification, due to the presence of a residual red component, which should be considered. A partial simplification could come from a sample filtration before performing the colorimetric analysis, not yet adopted in this study. In any case, this step is not considered sufficient to explain the complexity of the obtained data.

On the contrary, with regard to data monitored for juices (Table 5), L* values fall between 29.74 and 49.31, denoting a very wide range of lightness, maybe not only ascribable to contribution of the anthocyanins and ellagitannins, but also accounting for the sample turbidity. In any case, the smallest L* value corresponds to the highest a* value. This second parameter, ranging from 2.37 to 13.76, shows very high differences in the redness of the analyzed juices. Finally, the b* parameters fall between 1.28 and 5.79. Thus, when the ratio a*/b* is higher than 4 (DE, VK, SN, PA, WO), samples are perceived as basically red; when it is about 1–2 the perceived color is red-orange and for ratio a*/b*<1 (AC, BL, SZ) it appears orange-yellow. The attempt to match these results to the quantitative data obtained for anthocyanins and ellagitannins returns a good correlation between h_ab_ (color hue) and the ratio delphinidin/cyanidin expressed as sum of all the quantified glycosides (Figure S3).

To our best knowledge, only a few papers have been published on the use of pomegranate husks as natural mordant for dying [36,37], and just a few other papers deal with the CIEL*a*b* analysis of pomegranate fruit, external peels and arils [38], whereas no literature is available regarding the CIEL*a*b* analysis of pomegranate decoction. If our previous results [23,35] indicate that a correlation could be found between CIEL*a*b* parameters and bioactive compounds content of blueberry and goji berries, in which only one color component largely predominates, further studies are needed to better clarify if this fast, cheap and simple method could be used to predict the quali-quantitative content of bioactive molecules in juices and decoctions obtained from pomegranate. In juices of this fruit, in fact, two different components contribute in a quite different manner to the color development, whereas in decoctions, browning and precipitation processes could take place during the sample storage.

### 3.4. Spectrophotometric Determinations on Juices and Decoction

Collectively, decoction of pomegranate samples led to a high recovery of phenolic and flavonoid compounds compared to pomegranate juices. Values of Total Phenolic Content (TPC), expressed as mg of gallic acid equivalents (mg GAE) per gram of extract, ranged from 106.34 ± 0.42 to 263.05 ± 3.64 mg GAE/g after decoction of these varieties. The Total Flavonoid Content (TFC), expressed as rutin equivalents (mg RE) per gram of extract, varied from 18.63 ± 0.20 to 93.97 ± 0.39 mg RE/g. D-MV was the pomegranate decoction with the lowest values for both TPC and TFC. Conversely, the pomegranate decoctions of the other varieties were characterized by high values of TPC and also displayed comparable values of TFC.

Pomegranate juices showed TPC and TFC data about 20- and 300-fold inferior to decoctions. This trend was confirmed by their low antioxidant and chelating properties as assessed by six in vitro spectrophotometric assays (Table 6). Meanwhile, each decoction, regardless of variety, possessed better antioxidant and chelating abilities with respect to juices, with D-MV being the least interesting and D-SI the most potent. The latter was also characterized by high TPC and TFC values. Moreover, it can be extrapolated that pomegranate juices had limited anti-oxidative characteristics directly related to their low amount of phenols. Additionally, in this case, J-MV was one of the poorest varieties and J-SI was one of the best-in-class extracts. This general trend could account for the relatively inferior stability of this processed matrix (Table 6).

### 3.5. Enzymatic Assays

By analyzing the data regarding tyrosinase inhibition (expressed in terms of mg of kojic acid equivalent for gram of extract), it is possible to confirm the better potency of decoctions over juices; it should be noted that the D-MV sample, previously described as a low source of phenols and flavonoids, proved to be the most powerful among the juices. Pomegranate decoctions displayed tyrosinase inhibition ranging from 24.44 ± 1.45 to 118.50 ± 0.81 mg KAE/g, whereas pomegranate juices hardly reached 16.56 ± 0.85 mg KAE/g, with half of the considered varieties not active against this enzyme. These results highlight the presence of other bioactive components beyond ellagitannins which exert such an inhibitory activity on decoctions (Table S2).

The data on inhibition against α-amylase, a key enzyme affecting the availability of dietary glucose, were almost comparable among the pomegranate varieties for both decoctions and juices. Only slight differences can be highlighted, suggesting that pomegranate fruits of different varieties can be promising sources of functional ingredients characterized by a healthy panel of chemical and biological characteristics.

## 4. Conclusions

Fifteen varieties of pomegranate, some never studied so far, obtained from the same hatchery in the same harvesting year and having fruits with very different morphological characters, were evaluated. Their chemical composition was compared in terms of ellagitannins and anthocyanins, whose contents were evaluated both in juices and in peels, and in terms of polysaccharides detected only in peels. For the analysis of the two very different groups of phenolic compounds, we proposed a single HPLC method, thus providing an easy approach for the quality control of pomegranate. The resultant chromatographic profiles for both ellagitannin and anthocyanin fractions were qualitatively very similar and, in some cases, almost superimposable. At the same time, according to the noticeable morphological differences among the selected varieties, a wide variability in terms of total content of ellagitannins, anthocyanins and polysaccharides was highlighted. CIEL*a*b* colorimetry, suitable to analyze juices and decoctions as such, showed a good correlation with the ratio between the sum of delphinidin glycosides and sum of cyanidin glycosides, quantified in juices by HPLC–DAD analysis. Concerning the polysaccharides, a simple fractionation method suitable to collect three main polysaccharide fractions from each sample was applied. A preliminary analysis by size exclusion chromatography, applied to four varieties selected for their significant differences in the morphological characters, identified in all the samples a large predominance of polysaccharides with apparent molecular weight close to two million Dalton. Overall, the quantitative results indicate a wide variability in terms of ellagitannins and anthocyanin amount, also confirmed by colorimetric analysis. The Total Phenolic Content was low in pomegranate juices, and consistently higher in the decoctions. Different in vitro methods allowed acquisition of useful data on the antioxidant potency of juices and decoctions. Regarding the enzymatic tests, the anti-α-amylase activity was quite comparable among all the samples, while decoctions were generally much more active as anti-tyrosinase agents.

## Figures and Tables

**Table 1:** Characteristics of the fruit samples provided by the producer. na: not available; peel means exocarp + mesocarp; Sept, September; Oct, October.

Variety	Peel Thickness (cm)	Peel Weight (%)	Peel Color	Arils Weight (%)	Arils’ Color	Seeds’ Hardness	Ripening Date	Brix Sept 27^th^	Brix Oct 3^rd^	Brix Oct 11^th^	Brix Oct 17^th^	Brix Oct 25^th^	Taste
Acco (AC)	0.40	44.9	Red	55.1	Red	Soft	Sept 27^th^	16.2	19.2	-	-	-	sweet
Ariana (AR)	0.50	45.0	Red	55.0	Red	Soft	Oct 11^th^	-	15.2	16.5	-	-	bitter-sweet
Austin (AU)	0.25	41.6	Red+	58.4	Red+	Medium	Sept 27^th^	18.5	17.0	-	-	-	sweet
Black (BL)	0.25	47.5	Black	52.5	White	Hard	na	-	-	-	-	-	bitter
Desertnyi (DE)	0.25	49.8	Red-	50.2	Red	Soft	Oct 11^th^	-	16.0	17.0	-	-	bitter-sweet
Grossa di Faenza (GF)	0.80	54.7	Pink	45.3	Pink	Hard	Oct 17^th^	-	-	-	17.0	-	bitter-sweet
Mollar de Elche (ME)	0.50	35.5	Yellow/Pink	64.5	Pink	Soft+	Oct 17^th^	16.8	-	-	18.0	-	sweet
Medovyi Vasha (MV)	0.35	50.8	Red	49.2	Red	Soft	Oct 11^th^	-	-	15.5	-	-	sweet
Parfianka (PA)	0.45	46.0	Red	54.0	Red	Soft	Oct 17^th^	-	-	17.5	-	-	bitter-sweet
Provenza Francia (PF)	0.60	48.6	Pink	54.1	Pink	Hard	na	-	-	-	16.0	-	bitter-sweet
Sirenevyi (SI)	0.60	49.0	Orange/Red	51.0	Red	Soft	Oct 17^th^	-	13.2	14.8	16.0	-	sweet
Soft Seed Maule^®^ 116/17 (SM)	0.50	52.8	Red+	47.2	Red	Medium	Oct 17^th^	-	-	17.0	-	-	bitter-sweet
Shirin Zigar (SZ)	0.70	68.0	Red+	32.0	Red	Hard+	na	-	15.0	-	16.0	-	sweet
Vkusnyi (VK)	0.50	44.8	Red	55.2	Red	Soft	Oct 11^th^	-	17.5	-	-	-	bitter-sweet
Wonderful (WO)	0.50	44.7	Red	55.3	Red	Medium	Oct 25^th^	-	-	-	-	18.5	bitter-sweet

**Table 2:** Main phenolic compounds evaluated in the produced extracts for decoctions, with data expressed as mg/g. Punic., punicalagin; cya-3,5-diglc, cyanidin-3,5-*O*-diglucoside; del-3-glc, delphinidin-3-*O*-glucoside; cya-glc, cyanidin-3-*O*-glucoside; pel-glc, pelargonidin-3-*O*-glucoside; n.d.: not detected.

Decoctions	Punic. der-1	Punic. der-2	α-punic.	β-punic.	Punic. der-3	Ellagic der-1	Ellagic acid	Ellagic der-2	Ellagic der-3	Ellagic der-4	Ellagic der-5	cya-3,5-diglc	del-3-glc	cya-glc	pel-glc
Acco	2.65	14.67	17.65	46.01	1.60	0.78	0.29	3.00	0.47	0.08	0.04	0.09	0.03	0.03	n.d.
Ariana	2.48	47.57	3.82	8.98	0.97	0.34	0.07	1.42	0.49	0.06	0.03	0.05	n.d.	0.04	n.d.
Austin	3.11	11.88	12.77	33.07	1.43	1.10	0.19	1.76	0.45	0.07	0.09	0.41	0.16	0.15	0.05
Black	4.26	13.35	30.97	74.28	3.09	1.80	0.25	0.13	0.60	2.45	1.21	0.32	0.56	0.12	n.d.
Desertnyi	1.59	4.68	10.81	26.11	1.31	0.35	0.07	0.96	0.38	0.07	0.10	n.d.	n.d.	0.03	n.d.
Grossa di Faenza	2.30	7.41	13.41	34.95	1.43	0.85	0.17	1.91	0.49	0.06	0.09	n.d.	n.d.	0.03	n.d.
Mollar de Elche	3.65	13.92	18.18	38.80	1.16	1.21	0.41	3.05	0.56	0.02	0.02	n.d.	n.d.	n.d.	n.d.
Medovyi Vasha	2.17	5.80	7.10	17.50	0.67	0.34	0.07	1.22	0.31	0.09	0.04	n.d.	0.03	n.d.	0.05
Parfianka	2.40	5.82	9.41	28.51	1.77	0.48	0.14	0.96	0.32	0.06	0.05	0.03	0.00	0.03	0.00
Provenza Francia	2.53	8.16	21.73	52.69	2.00	0.90	0.48	2.53	0.53	0.07	0.06	n.d.	n.d.	n.d.	n.d.
Sirenevyi	2.75	11.10	17.54	0.44	2.17	0.81	0.34	1.57	0.38	0.04	0.04	0.03	0.03	0.03	n.d.
Soft Seed Maule^®^ 116/17	2.60	6.93	12.03	32.68	1.19	0.69	0.09	1.18	0.27	0.04	0.03	0.41	0.05	0.33	n.d.
Shirin Zigar	1.16	5.48	7.44	17.44	0.54	0.56	0.10	1.54	0.40	0.04	0.03	0.08	0.04	0.06	0.00
Vkusnyi	1.96	5.86	13.18	32.78	1.02	0.40	0.07	0.96	0.25	0.04	0.03	0.05	n.d.	0.07	n.d.
Wonderful	2.50	7.04	19.37	50.88	1.22	1.36	0.34	1.89	0.39	0.04	0.03	0.05	0.04	0.05	n.d.

**Table 3:** Main phenolic compounds evaluated in the produced extracts for juices diluted 1:1 *v/v* with ethanol, with data expressed as mg/L; Punic., punicalagin; del-3,5-diglc, delphinidin-3,5-*O*-diglucoside; cya-3,5-diglc, cyanidin-3,5-*O*-diglucoside; del-3-glc, delphinidin-3-*O*-glucoside; cya-glc, cyanidin-3-*O*-glucoside; pel-glc, pelargonidin-3-*O*-glucoside; antho der, anthocyanin derivative; n.d.: not detected.

Juices	α-punic	β-punic	Punic. der-3	Ellagic der-1	Ellagic acid	Ellagic der-2	Ellagic der-3	Ellagic der-4	Ellagic der-5	del-3,5-diglc	cya-3,5-diglc	del-3-glc	cya-glc	pel-glc	antho der
Acco	n.d.	n.d.	n.d.	n.d.	1.19	1.67	0.73	0.33	0.98	n.d.	93.34	8.64	61.99	5.14	1.46
Ariana	n.d.	n.d.	n.d.	n.d.	0.37	5.21	1.33	1.73	n.d.	77.10	65.07	82.00	81.48	7.18	3.43
Austin	n.d.	n.d.	n.d.	n.d.	0.24	1.35	0.53	n.d.	n.d.	n.d.	19.09	24.63	64.55	8.39	4.51
Black	11.07	11.64	4.62	2.15	0.27	4.66	3.86	0.61	2.37	n.d.	n.d.	n.d.	n.d.	n.d.	n.d.
Desertnyi	n.d.	n.d.	n.d.	n.d.	0.19	0.30	0.10	0.20	n.d.	37.10	34.07	46.00	52.43	7.97	4.80
Grossa di Faenza	n.d.	n.d.	n.d.	n.d.	0.69	1.18	0.73	0.80	n.d.	9.07	14.29	7.65	12.97	2.41	1.36
Mollar de Elche	n.d.	n.d.	n.d.	n.d.	0.20	1.37	0.31	n.d.	n.d.	n.d.	56.54	12.84	54.14	13.69	2.97
Medovyi Vasha	n.d.	n.d.	n.d.	n.d.	0.10	4.30	0.41	n.d.	n.d.	27.00	69.00	29.30	80.60	4.17	2.55
Parfianka	n.d.	n.d.	n.d.	n.d.	0.38	1.28	0.45	n.d.	n.d.	31.71	35.55	48.49	61.37	6.63	2.55
Provenza Francia	n.d.	n.d.	n.d.	n.d.	0.16	1.41	0.33	n.d.	n.d.	5.44	11.26	7.48	11.95	1.00	0.97
Sirenevyi	n.d.	n.d.	n.d.	n.d.	0.12	0.15	0.33	0.51	n.d.	53.99	52.90	53.12	68.90	10.59	7.11
Soft Seed Maule^®^ 116/17	n.d.	n.d.	n.d.	n.d.	0.63	1.70	0.58	0.48	n.d.	195.30	177.77	116.89	94.14	13.65	12.57
Shirin Zigar	n.d.	n.d.	n.d.	n.d.	0.59	2.17	0.76	n.d.	n.d.	n.d.	23.10	3.49	21.77	2.54	1.04
Vkusnyi	n.d.	n.d.	n.d.	n.d.	0.48	1.49	0.61	n.d.	n.d.	86.94	74.57	84.31	94.07	12.66	9.27
Wonderful	n.d.	n.d.	n.d.	n.d.	1.02	1.82	1.60	n.d.	n.d.	196.36	177.16	125.26	109.27	18.98	15.79

**Table 4:** Polysaccharide yields (%) of the three obtained fractions for each variety (D-P1, D-P2 and D-P3). Results are the mean of three independent determinations. The RSD value, evaluated on triplicates, was < 5%.

Variety	Fractions			Total
	D-P1 (%)	D-P2 (%)	D-P3 (%)	
Acco	0.43	8.80	1.98	11.21
Ariana	0.28	8.81	1.47	10.56
Austin	0.60	4.60	1.65	6.85
Black	2.61	8.91	0.83	12.35
Desertnyi	0.54	3.84	2.40	6.78
Grossa di Faenza	1.80	6.18	2.34	10.32
Mollar de Elche	0.40	3.35	4.62	8.37
Medovyi Vasha	1.21	2.04	4.75	8.00
Parfianka	2.02	2.38	3.48	7.88
Provenza Francia	1.23	1.50	1.04	3.77
Sirenevyi	0.31	6.96	1.11	8.38
Soft Seed Maule^®^ 116/17	1.09	5.73	1.54	8.36
Shirin Zigar	0.28	1.37	1.60	3.25
Vkusnyi	0.97	5.25	1.06	7.28
Wonderful	2.00	6.26	0.60	8.86

**Table 5:** Colorimetric data from CIEL*a*b* measurement of the pomegranate juices and decoctions. Results are reported as mean ± SD from three independent determinations.

**Decoctions**	L*	a*	b*	C*_ab_	**h_ab_**
D-AC	44.67 ± 0.10	1.57 ± 0.03	9.92 ± 0.09	10.04 ± 0.09	81.02 ± 0.10
D-AR	43.96 ± 0.63	2.11 ± 0.38	8.47 ± 0.87	8.73 ± 0.93	76.08 ± 1.03
D-AU	40.29 ± 0.10	7.60 ± 0.21	14.34 ± 0.36	16.23 ± 0.42	62.08 ± 0.10
D-BL	40.18 ± 1.46	2.98 ± 0.64	2.54 ± 0.24	3.92 ± 0.65	40.87 ± 3.56
D-DE	46.37 ± 0.52	1.30 ± 0.38	8.81 ± 1.53	8.90 ± 1.57	81.73 ± 1.13
D-GF	46.92 ± 0.20	0.70 ± 0.12	8.70 ± 0.63	8.73 ± 0.63	85.42 ± 0.45
D-ME	43.51 ± 0.17	6.41 ± 0.19	23.48 ± 0.50	24.34 ± 0.53	74.72 ± 0.13
D-MV	45.71 ± 0.21	1.22 ± 0.18	9.85 ± 0.91	9.93 ± 0.93	82.96 ± 0.39
D-PA	43.94 ± 0.51	4.41 ± 0.22	19.35 ± 0.73	19.84 ± 0.76	77.15 ± 0.16
D-PF	47.64 ± 0.66	1.61 ± 0.21	22.21 ± 1.14	22.27 ± 1.15	85.87 ± 0.32
D-SI	45.11 ± 0.49	5.04 ± 0.34	23.22 ± 1.02	23.76 ± 1.07	77.76 ± 0.30
D-SM	43.55 ± 0.19	10.92 ± 0.07	18.17 ± 0.09	21.20 ± 0.10	58.49 ± 0.45
D-SZ	42.31 ± 0.22	3.62 ± 0.15	14.17 ± 0.45	14.62 ± 0.47	75.66 ± 0.22
D-VK	46.85 ± 1.15	4.69 ± 0.21	21.53 ± 0.53	22.04 ± 0.55	77.72 ± 0.28
D-WO	44.73 ± 0.01	5.81 ± 0.08	19.94 ± 0.18	20.78 ± 0.19	73.74 ± 0.09
**Juices**	L*	a*	b*	C*_ab_	**h_ab_**
J-AC	40.91 ± 0.83	2.37 ± 0.18	3.28 ± 0.14	4.05 ± 0.22	54.19 ± 0.98
J-AR	35.02 ± 0.95	4.90 ± 0.23	1.49 ± 0.08	5.12 ± 0.25	16.92 ± 0.21
J-AU	38.28 ± 1.65	4.69 ± 0.69	3.55 ± 0.24	5.89 ± 0.69	37.32 ± 2.17
J-BL	45.96 ± 0.19	4.43 ± 0.10	5.00 ± 0.08	6.68 ± 0.13	48.47 ± 0.28
J-DE	37.02 ± 1.86	11.19 ± 0.96	1.65 ± 0.07	11.32 ± 0.96	8.44 ± 0.55
J-GF	49.31 ± 0.10	6.20 ± 0.10	5.51 ± 0.09	8.30 ± 0.14	41.60 ± 0.17
J-ME	35.02 ± 0.11	4.84 ± 0.13	4.77 ± 0.12	6.76 ± 0.14	44.35 ± 0.34
J-MV	39.47 ± 0.69	4.94 ± 0.34	1.37 ± 0.05	5.12 ± 0.34	15.56 ± 0.69
J-PA	36.53 ± 1.22	8.12 ± 0.77	1.77 ± 0.04	8.31 ± 0.76	12.36 ± 0.85
J-PF	44.65 ± 0.13	10.19 ± 0.14	5.79 ± 0.10	11.72 ± 0.17	29.63 ± 0.14
J-SI	47.34 ± 0.98	4.33 ± 0.54	2.04 ± 0.11	4.79 ± 0.49	25.46 ± 2.84
J-SM	29.74 ± 0.29	13.76 ± 0.35	2.57 ± 0.21	14.00 ± 0.38	10.56 ± 0.59
J-SZ	38.40 ± 1.28	2.55 ± 0.37	4.23 ± 0.34	4.94 ± 0.48	59.98 ± 1.73
J-VK	30.86 ± 0.13	8.02 ± 0.08	1.28 ± 0.08	8.13 ± 0.08	9.04 ± 0.48
J-WO	42.05 ± 1.63	6.98 ± 0.91	1.65 ± 0.09	7.18 ± 0.91	13.38 ± 0.84

**Table 6:** All values are reported as mean ± SD of three independent experiments. GAE: Gallic acid equivalent; RE: Rutin equivalent; TE: Trolox equivalent; EDTAE: EDTA equivalent; na: not active. Different letters in the same column indicate significant differences in the extracts (*p* > 0.05).

**Decoctions**	**Total Phenolic Content (mg GAE/g)**	**Total Flavonoid Content (mg RE/g)**	**DPPH (mmol TE/g)**	**ABTS (mmol TE/g)**	**CUPRAC (mmol TE/g)**	**FRAP (mmol TE/g)**	**Metal Chelating (mg EDTAE/g)**	**Phosphomolybdenum (mmol TE)**
D-AC	259.14 ± 0.47 ^ab^	93.97 ± 0.39 ^a^	3.91 ± 0.01 ^a^	4.79 ± 0.10 ^a^	7.63 ± 0.30 ^b^	6.93 ± 0.10 ^a^	31.22 ± 1.93 ^ab^	5.79 ± 0.13 ^d^
D-AR	213.22 ± 1.45 ^h^	61.15 ± 0.07 ^g^	3.68 ± 0.04 ^d^	3.45 ± 0.01 ^d^	5.24 ± 0.04 ^g^	4.60 ± 0.02 ^gh^	13.07 ± 2.81 ^de^	4.31 ± 0.26 ^f^
D-AU	242.79 ± 1.81 ^e^	66.51 ± 0.72 ^f^	3.88 ± 0.01 ^a^	4.26 ± 0.23 ^bc^	6.03 ± 0.013 ^de^	5.38 ± 0.06 ^f^	21.08 ± 0.90 ^c^	5.30 ± 0.13 ^e^
D-BL	254.91 ± 1.19 ^bc^	80.53 ± 1.52 ^c^	3.89 ± 0.01 ^a^	4.78 ± 0.14 ^a^	7.33 ± 0.09 ^c^	6.16 ± 0.09 ^c^	16.61 ± 2.64 ^cd^	6.53 ± 0.18 ^c^
D-DE	231.19 ± 1.40 ^f^	52.11 ± 0.30 ^h^	3.82 ± 0.01 ^b^	4.31 ± 0.12 ^bc^	5.90 ± 0.10 ^ef^	4.73 ± 0.04 ^g^	16.24 ± 1.95 ^cd^	5.35 ± 0.17 ^e^
D-GF	251.84 ± 0.30 ^cd^	77.32 ± 0.61 ^d^	3.89 ± 0.01 ^a^	4.73 ± 0.19 ^a^	7.13 ± 0.05 ^c^	5.97 ± 0.08 ^d^	17.58 ± 4.37 ^cd^	6.28 ± 0.25 ^c^
D-ME	258.26 ± 4.03^b^	74.22 ± 0.50 ^e^	3.88 ± 0.02 ^a^	4.84 ± 0.10 ^a^	7.83 ± 0.04 ^b^	6.17 ± 0.08 ^c^	34.69 ± 0.96 ^a^	7.32 ± 0.19 ^b^
D-MV	106.34 ± 0.42 ^k^	18.63 ± 0.20 ^l^	1.46 ± 0.01 ^g^	1.41 ± 0.06 ^g^	2.08 ± 0.02 ^j^	3.19 ± 0.01 ^i^	20.71 ± 1.82 ^c^	4.20 ± 0.18 ^f^
D-PA	129.27 ± 0.41 ^j^	39.87 ± 0.71 ^i^	1.43 ± 0.02 ^g^	1.37 ± 0.11 ^g^	2.57 ± 0.03 ^i^	2.58 ± 0.03 ^j^	2.98 ± 0.16 ^gh^	3.65 ± 0.15 ^g^
D-PF	226.43 ± 1.37 ^g^	52.67 ± 0.16^h^	3.74 ± 0.02 ^c^	3.49 ± 0.30 ^c^	5.72 ± 0.01 ^f^	4.44 ± 0.13 ^h^	15.88 ± 2.65 ^cd^	5.48 ± 0.11 ^de^
D-SI	263.05 ± 3.64^a^	83.63 ± 0.21^b^	3.89 ± 0.01^a^	4.61 ± 0.16^a^	8.09 ± 0.10 ^a^	6.75 ± 0.08 ^b^	28.24 ± 1.19 ^b^	7.78 ± 0.16 ^a^
D-SM	148.14 ± 2.14^i^	31.69 ± 0.06^k^	1.83 ± 0.06^f^	1.43 ± 0.18^f^	3.01 ± 0.05 ^h^	2.49 ± 0.01 ^j^	10.27 ± 0.81 ^ef^	3.60 ± 0.11 ^g^
D-SZ	233.75 ± 1.66^f^	52.61 ± 0.71^h^	3.83 ± 0.02^b^	4.20 ± 0.11^b^	6.20 ± 0.07^d^	4.64 ± 0.04^g^	16.50 ± 2.80 ^cd^	5.08 ± 0.11 ^e^
D-VK	147.92 ± 0.51^i^	36.05 ± 0.42^j^	1.91 ± 0.01^e^	2.05 ± 0.07^e^	3.06 ± 0.02^h^	2.32 ± 0.01^k^	12.54 ± 3.20 ^de^	3.54 ± 0.12 ^g^
D-WO	248.93 ± 2.98^d^	61.42 ± 0.45^g^	3.91 ± 0.01^a^	4.02 ± 0.31^a^	7.30 ± 0.21^c^	5.71 ± 0.08^e^	14.16 ± 0.43 ^de^	6.44 ± 0.06 ^c^
**Juices**	**Total Phenolic Content (mg GAE/g)**	**Total Flavonoid Content (mg RE/g)**	**DPPH (mmol TE/g)**	**ABTS (mmol TE/g)**	**CUPRAC (mmol TE/g)**	**FRAP (mmol TE/g)**	**Metal Chelating (mg EDTAE/g)**	**Phosphomolybdenum (mmol TE)**
J-AC	11.56 ± 0.01 ^pq^	0.38 ± 0.04 ^n^	0.05 ± 0.01 ^mn^	0.09 ± 0.01 ^i^	0.20 ± 0.01	0.13 ± 0.01 ^mn^	4.04 ± 0.89 ^gh^	1.15 ± 0.07 ^hijk^
J-AR	16.30 ± 0.17 ^mno^	0.52 ± 0.08 ^n^	0.12 ± 0.01 ^jk^	0.14 ± 0.01 ^h^	0.26 ± 0.01 ^l^	0.24 ± 0.01 ^lmn^	na	1.06 ± 0.03 ^ijkl^
J-AU	18.01 ± 0.07 ^mn^	0.36 ± 0.04 ^n^	0.11 ± 0.01 ^jkl^	0.17 ± 0.01 ^g^	0.28 ± 0.01 ^l^	0.23 ± 0.01 ^lmn^	3.14 ± 0.08 ^gh^	0.88 ± 0.13 ^klm^
J-BL	14.77 ± 0.02 ^mnop^	0.63 ± 0.02 ^m^	0.15 ± 0.01 ^ij^	0.14 ± 0.01 ^h^	0.27 ± 0.01 ^l^	0.34 ± 0.01 ^l^	na	0.28 ± 0.03 ^n^
J-DE	17.83 ± 0.01 ^mn^	0.61 ± 0.02 ^m^	0.13 ± 0.01 ^jk^	0.15 ± 0.01 ^h^	0.29 ± 0.01 ^l^	0.28 ± 0.01 ^lm^	4.89 ± 0.34 ^gh^	0.71 ± 0.08 ^lmn^
J-GF	18.44 ± 0.16 ^m^	0.32 ± 0.05 ^n^	0.07 ± 0.01 ^lm^	0.11 ± 0.01 ^i^	0.26 ± 0.01 ^l^	0.21 ± 0.01 ^lmn^	2.13 ± 0.45 ^gh^	0.88 ± 0.05 ^klm^
J-ME	8.20 ± 0.06 ^q^	0.22 ± 0.01 ^o^	0.02 ± 0.01 ^n^	0.05 ± 0.01 ^j^	0.16 ± 0.01 ^n^	0.10 ± 0.01 ^n^	1.71 ± 0.30 ^gh^	1.55 ± 0.16 ^h^
J-MV	12.73 ± 0.08 ^op^	0.32 ± 0.07 ^n^	0.07 ± 0.01 ^lm^	0.10 ± 0.01 ^i^	0.22 ± 0.01 ^m^	0.16 ± 0.01 ^mn^	2.48 ± 0.02 ^gh^	1.46 ± 0.02 ^hi^
J-PA	14.88 ± 0.05 ^monp^	0.33 ± 0.03 ^n^	0.10 ± 0.01 ^jkl^	0.13 ± 0.01 ^i^	0.26 ± 0.01 ^l^	0.22 ± 0.01 ^lmn^	4.40 ± 0.46 ^gh^	1.04 ± 0.09 ^jkl^
J-PF	14.06 ± 0.52 ^mnop^	0.44 ± 0.04 ^n^	0.12 ± 0.01 ^jk^	0.12 ± 0.01 ^i^	0.24 ± 0.01 ^m^	0.24 ± 0.01 ^lmn^	4.27 ± 1.25 ^gh^	0.59 ± 0.04 ^mn^
J-SI	13.88 ± 0.08 ^nop^	0.34 ± 0.07 ^n^	0.09 ± 0.01 ^klm^	0.12 ± 0.01 ^i^	0.24 ± 0.01 ^m^	0.15 ± 0.01 ^mn^	5.69 ± 0.63 ^fg^	1.34 ± 0.04 ^hij^
J-SM	12.66 ± 0.13 ^opq^	0.33 ± 0.08 ^n^	0.10 ± 0.01 ^jkl^	0.11 ± 0.01 ^i^	0.23 ± 0.01 ^m^	0.17 ± 0.01 ^lmn^	na	0.83 ± 0.03 ^klm^
J-SZ	16.52 ± 0.08 ^mno^	0.44 ± 0.03 ^n^	0.12 ± 0.01 ^jk^	0.16 ± 0.01 ^g^	0.28 ± 0.01 ^l^	0.19 ± 0.01 ^lmn^	19.67 ± 1.34 ^c^	0.48 ± 0.02 ^mn^
J-VK	23.54 ± 0.22 ^l^	0.28 ± 0.09 ^n^	0.22 ± 0.01 ^h^	0.20 ± 0.01 ^g^	0.37 ± 0.01 ^k^	0.28 ± 0.01 ^lm^	3.15 ± 0.05 ^gh^	0.86 ± 0.05 ^klm^
J-WO	18.31 ± 0.23 ^mn^	0.35 ± 0.02 ^n^	0.20 ± 0.01 ^hi^	0.18 ± 0.01 ^g^	0.31 ± 0.01 ^k^	0.27 ± 0.01 ^lm^	4.17 ± 0.71 ^gh^	1.05 ± 0.07 ^jkl^

## Supplementary Materials

The following are available online at https://www.mdpi.com/2076-3921/9/3/238/s1. **Figure S1**: HPLC–DAD profiles at 380, 370 and 520 nm of the decoction from Black and of the juice from Wonderful varieties. **Figure S2**: Hydrodynamic volume of the main polysaccharides fractions obtained by SEC for the varieties Acco, Wonderful, Black, Mollar de Elche. **Figure S3**: Correlation between h_ab_ (color hue) and the delphinidin/cyanidin ratio, with both delphinidin and cyanidin expressed as sum of all the quantified glycosides in the fresh juices. **Table S1:** Main molecules identified by HPLC-MS in pomegranate samples. **Table S2**: α-Amylase and tyrosinase inhibition of all juices and decoctions.
