# Supplementary material for: Characterization of Arils Juice and Peel Decoction of Fifteen Varieties of Punica granatum L.: A Focus on Anthocyanins, Ellagitannins and Polysaccharides
Source: Antioxidants (Basel). 2020 Mar 13;9(3):238. doi: 10.3390/antiox9030238 (PMC7139709; doi:10.3390/antiox9030238)

Table S1. Main molecules identified by HPLC-MS in pomegranate samples.

**Ellagitannins**	**rt**	**[MH]$^-$**	**Identified compounds**
3	3.9	1083	α-punicalagin
4	5.9	1083	β-punicalagin
7	10.4	301	ellagic acid
**Anthocyanins**	**rt**	**[M]$^+$**	**Identified compounds**
Anthox	6.1	627	delphinidin-3,5-*O*-diglucoside
Antho1	6.8	611	cyanidin-3,5-*O*-diglucoside
Antho2	7.5	465	delphinidin-3-*O*-glucoside
Antho3	8.1	449	cyanidin-3-*O*-glucoside
Antho4	8.8	433	pelargonidin-3-*O*-glucoside

Table S2. α-Amylase and tyrosinase inhibition of all juices and decoctions.

**Decoctions**	**α-Amylase inhibition (mg ACAE/g)**	**Tyrosinase inhibition (mg KAE/g)**
**D-AC**	59.17±0.79^{cde}	51.12±1.59^{gh}
**D-AR**	74.49±0.63^{b}	40.99±1.46^{i}
**D-AU**	77.41±5.94^{b}	54.03±0.32^{fg}
**D-BL**	63.73±3.44^{cd}	76.24±1.25^{c}
**D-DE**	67.85±1.66^{bc}	62.86±0.45^{e}
**D-GF**	75.10±1.22^{b}	53.54±1.54^{fg}
**D-ME**	77.15±0.52^{b}	79.76±0.81^{c}
**D-MV**	57.95±0.49^{def}	118.50±0.81^{a}
**D-PS**	77.25±0.66^{b}	28.71±1.92^{j}
**D-PF**	56.70±0.90^{defg}	48.70±1.06^{h}
**D-SI**	75.98±3.40^{b}	86.06±0.39^{b}
**D-SN**	54.97±0.78^{defgh}	28.83±1.52^{j}
**D-SZ**	76.88±0.40^{b}	57.53±2.51^{f}
**D-VK**	52.95±0.41^{efghij}	24.44±1.45^{j}
**D-WO**	56.64±0.14^{defg}	70.80±2.31^{d}
**Juices**	**α-Amylase inhibition (mg ACAE/g)**	**Tyrosinase inhibition (mg KAE/g)**
**J-AC**	45.17±0.62^{hij}	na
**J-AR**	45.20±0.86^{hij}	na
**J-AU**	47.14±0.78^{ghij}	na
**J-BL**	133.64±14.06^{a}	na
**J-DE**	51.23±0.65^{efghij}	na
**J-GF**	49.50±1.54^{efghij}	na
**J-ME**	43.47±0.39^{j}	na
**J-MV**	47.24±1.21^{ghij}	na
**J-PA**	48.39±1.12^{fghij}	4.86±0.56^{l}
**J-PF**	53.06±1.15^{efghij}	7.63±1.37^{l}
**J-SI**	44.85±0.14^{ij}	8.41±1.13^{l}
**J-SN**	51.33±1.04^{efghij}	6.85±1.16^{l}
**J-SZ**	54.39±1.41^{defghi}	7.35±1.46^{l}
**J-VK**	77.09±0.96^{b}	16.56±0.85^{k}
**J-WO**	51.07±0.55^{efghij}	5.87±0.70^{l}

Values are reported as mean ± SD of three independent experiments. ACAE: Acarbose equivalent; KAE: Kojic acid equivalent; na: not active. Different letters in the same column indicate significant differences in the extracts ($p > 0.05$).

Figure S1. HPLC-DAD profiles at 380, 370 and 520 nm of the decoction from Black and of the juice from Wonderful varieties.

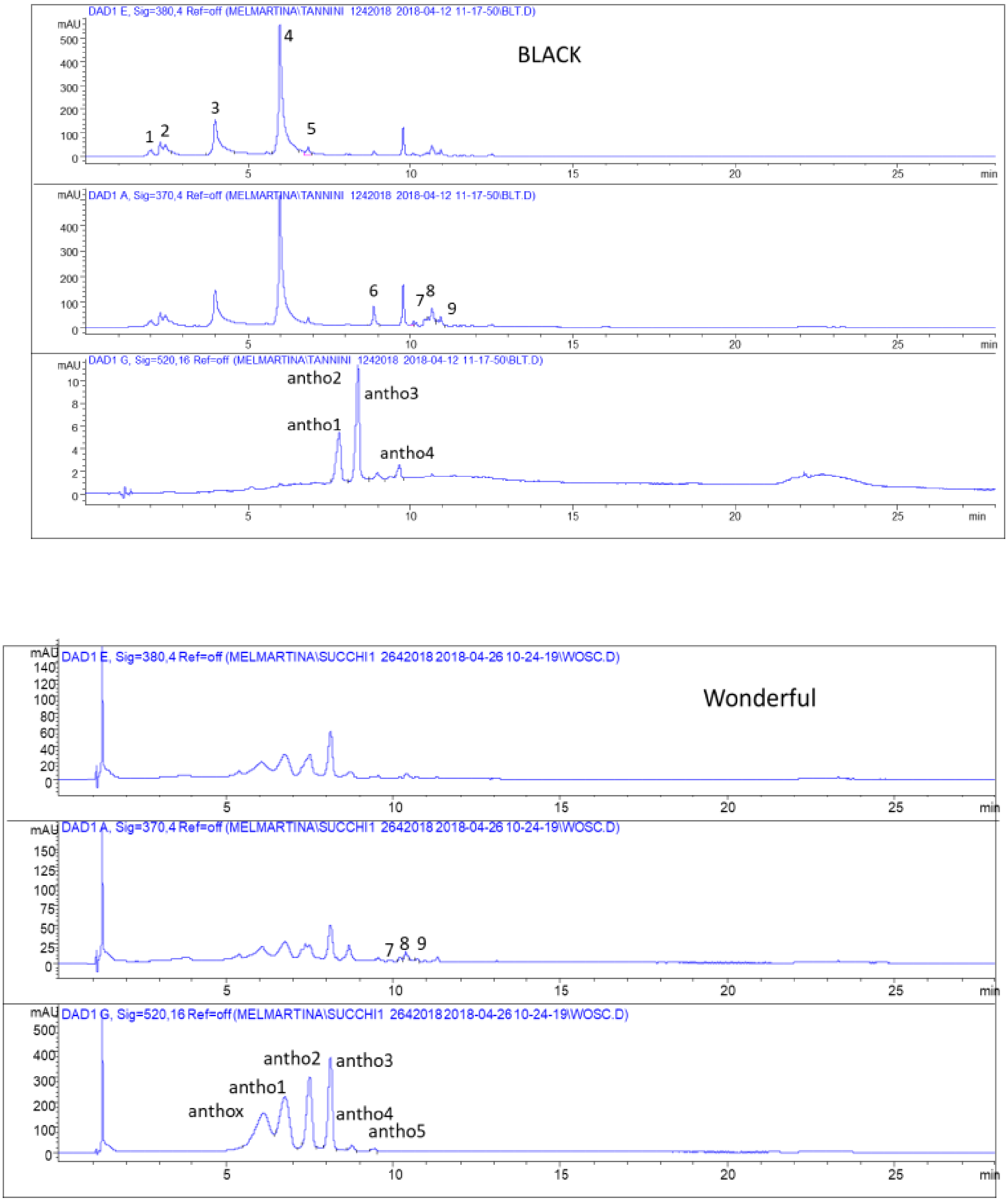

Peak n° **1**, **2**, **5**, **6**, **8**, **9**, **antho5**, unidentified; **3**, α-punicalagin; **4**, β-punicalagin; **7**, ellagic acid; **anthox**, delphinidin-3,5-*O*-diglucoside; **antho1**, cyanidin-3,5-*O*-diglucoside; **antho2**, delphinidin-3-*O*-glucoside; **antho3**, cyanidin-3-*O*-glucoside; **antho4**, pelargonidin-3-*O*-glucoside.

Figure S2. Hydrodynamic volume of the main polysaccharides fractions obtained by SEC for the varieties Acco, Wonderful, Black, Mollar de Elche.

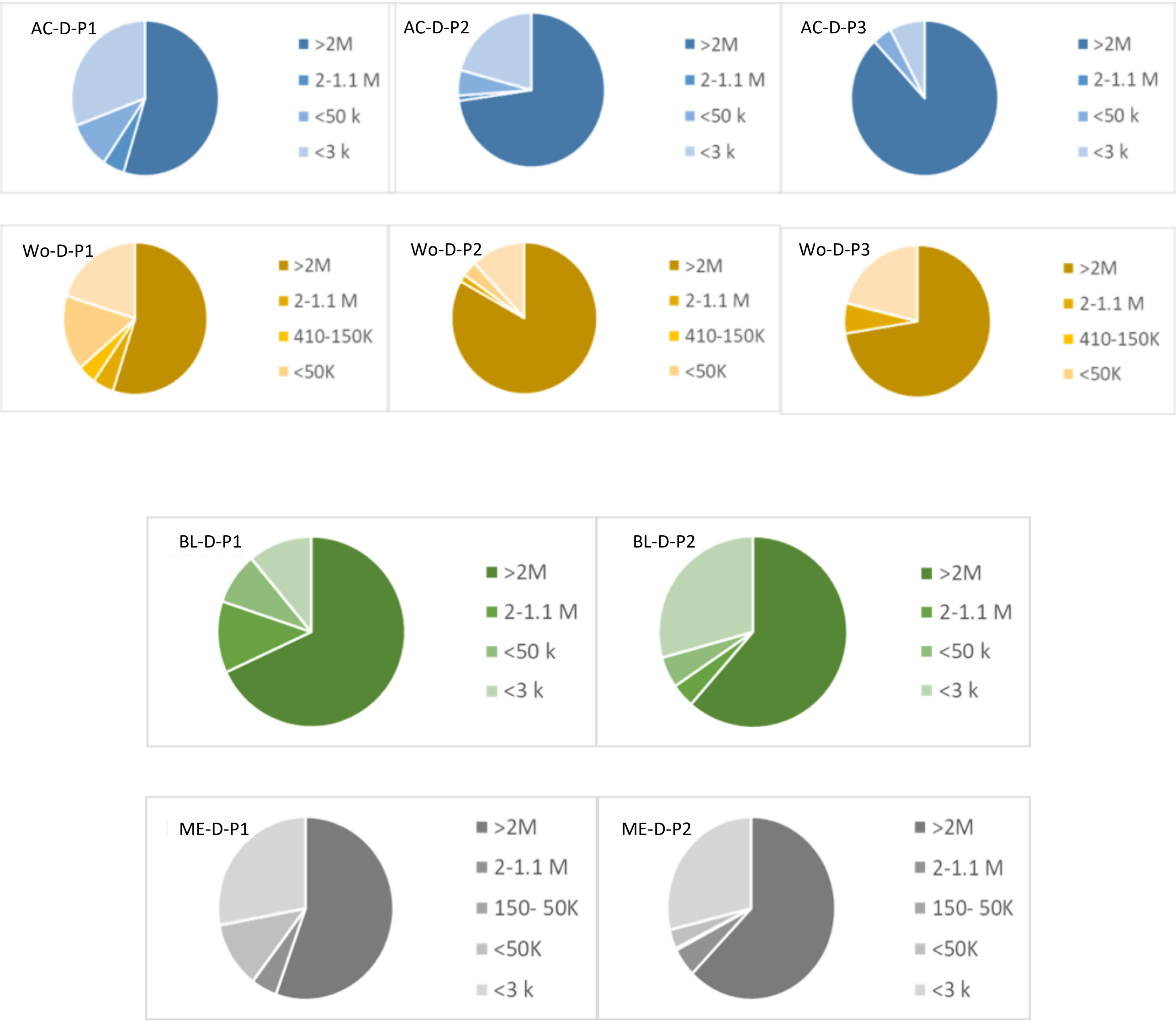

Figure S3. Correlation between h_{ab} (color hue) and the delphinidin/cyanidin ratio, with both delphinidin and cyanidin expressed as sum of all the quantified glycosides in the fresh juices.

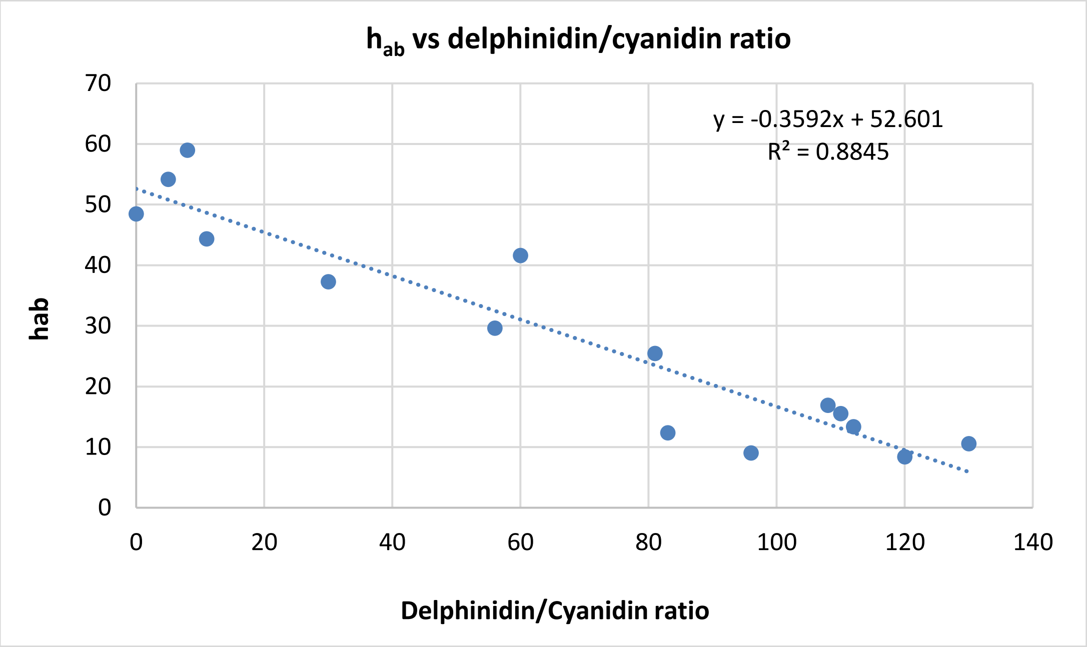

Supplement: Supplementary file 1 [file antioxidants-09-00238-s001.pdf]
